# *Aniba canelilla* (Kunth) Mez (Lauraceae) Essential Oil: Effects on Oxidative Stress and Vascular Permeability

**DOI:** 10.3390/antiox11101903

**Published:** 2022-09-26

**Authors:** Eloise K. Serrão Cardoso, Karen Kubota, Diandra Araújo Luz, Paulo Fernando S. Mendes, Pablo Luis B. Figueiredo, Rafael Rodrigues Lima, Cristiane S. Ferraz Maia, Enéas Andrade Fontes-Júnior

**Affiliations:** 1 Laboratory of Pharmacology of Inflammation and Behavior, Faculty of Pharmacy, Institute of Health Science, Federal University of Pará, Belém 66075-110, PA, Brazil; 2 Laboratory of Structural and Functional Biology, Institute of Biological Sciences, Federal University of Pará, Belém 66075-110, PA, Brazil; 3 Natural Products Chemistry Laboratory, State University of Pará, Belém 66050-540, PA, Brazil

**Keywords:** *Aniba canelilla*, essential oil, antioxidant, vascular permeability, inflammation, oxidative stress

## Abstract

The present study aimed to investigate the antioxidant activity of *Aniba canelilla* (kunth) Mez (Lauraceae) essential oil (AcEO), exploring its potential for prevention and/or treatment of oxidative stress and associated inflammatory process. With this aim, Wistar rats (*n* = 6/group) were pre-treated intraperitoneally with saline (0.9%) or AcEO (2 or 5 mg/kg) for 5 days. One hour after the last dose, inflammation and oxidative stress were induced by carrageenan (0.3 mg/kg; ip.) administration. Total antioxidant capacity, reduced glutathione (GSH) and lipid peroxidation levels, protein concentration, and leukocyte migration were evaluated in peritoneal fluid. Lipid peroxidation was also evaluated in plasma. Carrageenan strongly reduced the peritoneal antioxidant capacity and GSH concentration, increasing peritoneal and plasma lipid peroxidation. It also promoted increased plasma leakage and leukocyte migration. Treatment with AcEO (2 and 5 mg/kg), whose major constituent was 1-nitro-2-phenylethane (77.5%), increased the peritoneal antioxidant capacity and GSH concentrations, and reduced lipid peroxidation, both peritoneal and plasma, thus inhibiting the carrageenan-induced oxidative imbalance. AcEO also reduced the carrageenan-induced plasma leakage and leukocyte migration. These data demonstrate the AcEO antioxidant activity and its ability to modulate plasma leakage and leukocyte migration, confirming its potential for treating diseases associated with inflammation and oxidative stress.

## 1. Introduction

Free radicals, small molecules of unpaired free electrons, are largely produced during physiological metabolism and pathological processes. They perform important biological functions, such as defense against aggression, metabolism, energy production, fertilization, and gene activation [1,2], but they are also strongly associated with the genesis and evolution of many chronic and degenerative diseases [3,4]. Maintenance of homeostasis depends on a delicate balance between reactive species, primarily oxygen (ROS) and nitrogen (RNS), and systemic antioxidant mechanisms, which include enzymes such as Superoxide Dismutase (SOD), Catalase (CAT) and Glutathione Peroxidase (GPx), and non-enzymatic agents such as glutathione, alpha-tocopherol, and ascorbic acid [2,5].

Disruption of oxidative balance, caused by excessive production of ROS and RNS in combination or not with a decrease in enzymatic and non-enzymatic antioxidant activity, is known as oxidative stress. In this state, the oxidation process of biomolecules, such as lipids, proteins and nucleic acids, begins, generating functional and structural damages that compromise from cell activity and viability to functionality of tissues and organs, underlying which is the establishment of pathological conditions, usually chronic, such as inflammatory and degenerative diseases, obesity, diabetes, cardiovascular diseases, neoplasms, and neurological disorders [2,4].

The acquisition of such knowledge about oxidative stress influence on human illness has driven the search for strategies that promote systemic oxidative balance to prevent or treat pathological conditions. Ingestion of exogenous substances, mainly plant-derived products, including fruits, vegetables, cereals, flowers, or spices used for medicinal or food purposes, which scavenge free radicals, oxygen inhibitors and/or reducing agents, has shown great potential to decrease oxidative damage [6,7,8]. Aromatic plants, due to their secondary metabolites, such as polyphenols (phenolic acids, flavonoids, anthocyanins, lignans and stilbenes), carotenoids (xanthophylls and carotenes) and vitamins (vitamin E and C), demonstrate important antioxidant potential [6,9,10], in addition to anti-inflammatory, antibacterial, antiviral, antiaging and anticancer properties [11,12,13].

Within the great biodiversity and medicinal culture of the Amazon region, *Aniba canelilla* (Kunth) Mez [syn. *Aniba elliptica* A.C. Sm., *Cryptocarya canelilla* Kunth, and others], from Lauraceae family, an aromatic tree popularly known as ‘casca-preciosa’ (precious bark) or ‘falsa-canela’ (false cinnamon) stands out for its economic and medicinal potential [14,15]. Recognized for its characteristic cinnamon odor, attributed to its major constituent, 1-nitro-2-phenylethane (1N2F), *A. canelilla* is widely used in traditional medicine for the treatment of painful and inflammatory processes, in addition to gastrointestinal, neurological, and psychiatric disorders, and in the treatment of infections [16,17].

Given this potential, several studies have been conducted to verify the safety and efficacy of its use, having demonstrated the low toxicity of its essential oil (EO) (LD50 = 720 mg/kg, oral) and 1N2F (LD50 = 712 mg/kg, oral; and 490 mg/kg, ip.) in murine models [18,19,20]. Antinociceptive, anti-inflammatory [21,22,23,24,25,26], antihypertensive, bradycardic, anticonvulsant, anticholinesterase, anxiolytic and antibiotic activity against protozoa, fungi and bacteria have also been demonstrated [21,22,23,27,28,29,30]. Its antioxidant capacity has so far been verified only in physicochemical assays, in which its ability to scavenge DPPH (2,2-diphenyl-1-picrylhydrazyl) radicals was demonstrated [21]. In view of this, the present study aimed to investigate the antioxidant activity of *A. canelilla* essential oil (AcEO) in animal model, and to evaluate its application in prevention and/or treatment of oxidative stress and inflammatory processes correlated.

## 2. Materials and Methods

### 2.1. Chemicals and Reagents

Hexane (Chromatographic grade solvents), DTNB, TBA, ABTS, potassium persulfate and Trolox obtained from Sigma-Aldrich (Darmstadt, Germany). All chemicals used were of analytical grade. Indomethacin, Carrageenan Lambda C-3889, and Tween 80 (Sigma-Aldrich, St. Louis, MO, USA). Ketamine and Xylazine (Cristália, RJ, Brazil).

### 2.2. Animals

Male Wistar rats (*n* = 30), with an average weight of 150 g, from Federal University of Pará (UFPA) central vivarium, were kept in polypropylene cages (39 × 32 × 16 cm; up to 3/box) under standardized conditions of feeding and water (ad libitum), temperature (22 + 1 °C), exhaustion, and light/dark cycle (light from 6 a.m. to 6 p.m.). Experimental procedures were approved by UFPA’s Animal Research Ethics Committee (CEPAE; protocol No. 5320260521) and conducted in accordance with Care and Use of Laboratory Animals Guide (2011).

### 2.3. Plant Material, Extraction, and Analysis of Essential Oil

Leaves of a wild specimen of *A. canelilla* were collected in Belém, Pará state, Brazil (coordinates: 1°27′20.3″ S; 48°26′18.1″ W). Leaves were dried for seven days at room temperature, ground, and then submitted to EO extraction using a Clevenger-type apparatus (3 h) in triplicate. Then, oil was dried over anhydrous sodium sulfate, and the total oil yield was expressed as a percentual of the dried material. The specimen was collected in agreement with the Brazilian laws concerning the protection of biodiversity (SISGEN A704928).

EO was analyzed on a GCMS-QP2010 Ultra system (Shimadzu Corporation, Tokyo, Japan), equipped with an AOC-20i auto-injector and the GCMS-Solution system containing the Adams (2007) [31] and FFNSC 2 [32] libraries. A Rxi-5ms (30 m × 0.25 mm; 0.25 μm film thickness) silica capillary column (Restek Corporation, Bellefonte, PA, USA) was used. The conditions of analysis were: injector temperature of 250 °C; oven temperature programming of 60–240 °C (3 °C/min); helium as the carrier gas, adjusted to a linear velocity of 36.5 cm/s (1.0 mL/min); split mode injection for 1.0 μL of the sample (oil 3.0 μL: hexane 500 μL); split ratio 1:20; ionization by electronic impact at 70 eV; ionization source and transfer line temperatures of 200 and 250 °C, respectively. Mass spectra were obtained by automatically scanning every 0.3 s, with mass fragments in the range of 35–400 *m*/*z*. The retention index was calculated for all volatile components using a homologous series of C8-C40 n-alkanes (Sigma-Aldrich, St. Louis, MO, USA), according to the linear equation of van den Dool and Kratz (1963) [33]. Quantitative data regarding the volatile constituents were obtained by peak-area normalization using a GC 2010 Plus Series, coupled with an FID Detector, operated under similar conditions to the GC-MS system.

### 2.4. Drugs and Solutions

Carrageenan (0.3 mg/kg) and Indomethacin (10 mg/kg) solutions were prepared in saline (0.9%). AcEO (density = 1.020 g/mL) was prepared at doses of 2 and 5 mg/kg [20,26], being solubilized in sterile saline (0.9%), with the addition of tween 80 (1%). Drugs were administered intraperitoneally (ip.), with a standard volume of 0.1 mL/100 g of body weight.

### 2.5. Carrageenan-Induced Peritonitis

According to Souza and Ferreira (1985) [34] protocol, inflammation and consequent oxidative stress were induced in the peritoneal cavity by carrageenan (0.3 mg/kg) administration. For this, animals were randomly distributed into five groups (*n* = 6/group), which were previously treated for five days with saline (White and Carrageenan Groups), AcEO (2.0 or 5.0 mg/kg), or Indomethacin (10 mg/kg). Carrageenan was administered 1 h after the last pretreatment dose.

**Figure 1:**
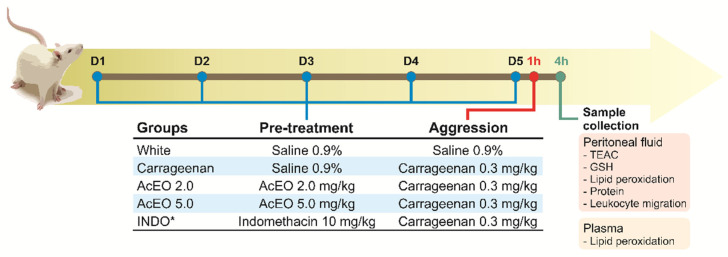
Experimental design. * Anti-inflammatory standard.

Four hours later, animals were euthanized under anesthesia (ketamine + xylazine; 91 + 9.1 mg/kg; ip.), after which 5 mL of saline were injected into peritoneal cavity, and the wash was collected to oxidative balance and leukocyte migration evaluation. Blood samples were also collected by cardiac puncture, and then centrifuged (3500 RPM for 10 min) for plasma separation, for the assessment of the oxidative balance (Figure 1).

### 2.6. Oxidative Biochemistry Assays

#### 2.6.1. Total Antioxidant Capacity

Trolox equivalent antioxidant capacity (TEAC) was evaluated through the reaction between ABTS and potassium persulfate (K_2_S_2_O_8_), producing the cationic radical ABTS^•+^ (3-ethylbenzothiazoline-6-sulfonate, diammonium salt), as proposed by Re et al. (1999) [35]. Antioxidant compounds reduce the formed radical back to ABTS, which can be measured by spectrophotometry (*λ* = 734 ηm). The AcEO ability to reduce radicals was evaluated 5 min from addition of 30 μL of sample in 2970 μL of ABTS^•+^. Results expressed as Trolox equivalent (μmol/mL).

#### 2.6.2. Reduced Glutathione Level (GSH)

Were evaluated their ability to reduce 5,5-dithiobis-2-nitrobenzoic acid (DTNB) to (TNB). First, 20 μL of sample, deproteinized with trichloroacetic acid (2%), was placed in 20 μL of distilled water and 3 mL of SOD buffer, and then read in a spectrophotometer (*λ* = 412 ηm) [36]. Then, 100 μL of DTNB was added, allowed to react for 3 min, and read again. GSH concentration was calculated using a standard curve and expressed in μg/mL.

#### 2.6.3. Lipid Peroxidation

It was evaluated from the reaction of polyunsaturated fatty acids metabolites, products of lipid peroxidation, with thiobarbituric acid, which produces measurable staining by spectrophotometry (*λ* = 535 nm) [37,38]. For this, 100 μL of the sample was mixed with 500 μL of thiobarbituric acid (10 ηM), and heated at 94 °C for 60 min in a water bath. The solution was cooled for 10 min, then 2 mL of l-butyl alcohol was added, followed by centrifugation (2500 rpm for 10 min) and collection of 1 mL of the supernatant, which was read in a spectrophotometer. Results were expressed as thiobarbituric acid reactive substances (TBARS) concentration, calculated using standard curve, in ηmol/mL.

### 2.7. Vascular Permeability Assays

#### 2.7.1. Protein Concentration

Doles total protein colorimetric kit was used, as proposed by Gornall (1949) [39]. In this way, 50 μL of peritoneal lavage was added to 2.5 mL of Biureto (0.114 M Trisodium Citrate; 0.21 M Sodium Carbonate, and 0.01 M Copper Sulfate). Two drops of sodium hydroxide (500 mmol/L) were then added, and reading was taken in a spectrophotometer (*λ* = 550 ηm). Bovine albumin (40 mg/mL) was used as standard, and results are expressed in mg/mL.

#### 2.7.2. Cell Migration Assessment

This was evaluated through the total leukocyte count in peritoneal lavage. For this, 20 µL of sample was diluted in 380 µL of Turk’s solution. Counting was performed in Neubauer chamber by optical microscopy (400× magnification). Results expressed as number of cells/mL.

### 2.8. Statistical Analysis

Data normality was assessed using the Shapiro–Wilk method. The difference between groups was verified by one-way ANOVA, followed by Dunnett’s test, when data presented Gaussian distribution; and Kruskal–Wallis test, followed by Dunn’s test, in other cases. Differences with *p* < 0.05 were considered statistically significant.

## 3. Results

### 3.1. Essential Oil Composition

The oil yield was 1.3 ± 0.1%. Benzenoid class (80.0 ± 5.8) were predominant in AcEO, and a low amount of oxygenated sesquiterpenes (6.9 ± 1.6%), sesquiterpene hydrocarbons (6.9 ± 4.7), monoterpene hydrocarbons (1.0 ± 0.9%), and oxygenated (3.5 ± 1.0%) were detected (Table 1).

**Figure 2:**
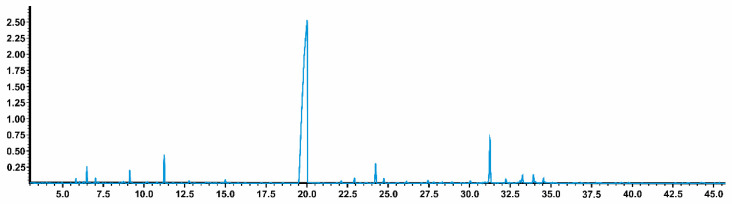
*Aniba canelilla* essential oil chromatogram.

Thirty-two compounds were identified, and the major compound was 1-nitro-2-phenylethane (1N2F), accounting for 77.5 ± 4.8%. Other compounds were detected in minor amounts, such as *E*-caryophyllene (3.6%), linalool (2.8 ± 0.7%), β-longipinene (1.6 ± 0.9%), and benzene acetaldehyde (1.1 ± 0.1%). The chromatograms of AcEO are shown in Figure 2.

### 3.2. AcEO Antioxidant Activity

#### 3.2.1. AcEO Improves Total Antioxidant Capacity

**Figure 3:**
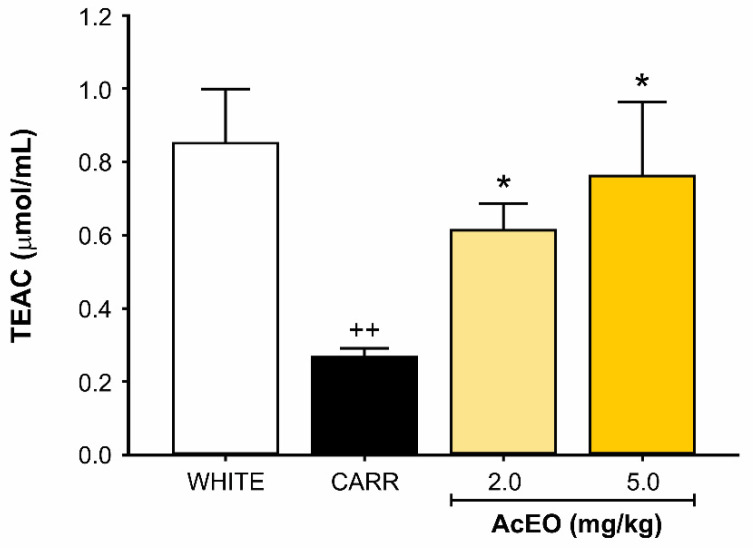
Effect of *Aniba canelilla* essential oil (AcEO; 2.0 and 5.0 mg/kg; ip.) on the total antioxidant capacity of the peritoneal fluid of rats submitted to carrageenan (CARR)-induced (0.3 mg/kg) peritonitis. Data expressed as mean ± SEM (*n* = 6/group). ^++^ *p* < 0.01 vs. white group; * *p* < 0.05 vs. CARR group. Kruskal–Wallis followed by Dunn’s test.

Intraperitoneal administration of carrageenan (0.3 mg/kg) significantly reduced the total antioxidant capacity of peritoneal fluid when compared to white group (0.27 ± 0.02 μmol/mL and 0.85 ± 0.15 μmol/mL, respectively; *p* < 0.01). Pretreatment with AcEO (2 and 5 mg/kg; ip.) was able to prevent this effect (*p* < 0.05), generating a result (0.61 ± 0.07 μmol/mL and 0.76 ± 0.20 μmol/mL, respectively) comparable to white group (*p* = 1.00 to both; Figure 3).

#### 3.2.2. AcEO Prevents GSH Decrease

**Figure 4:**
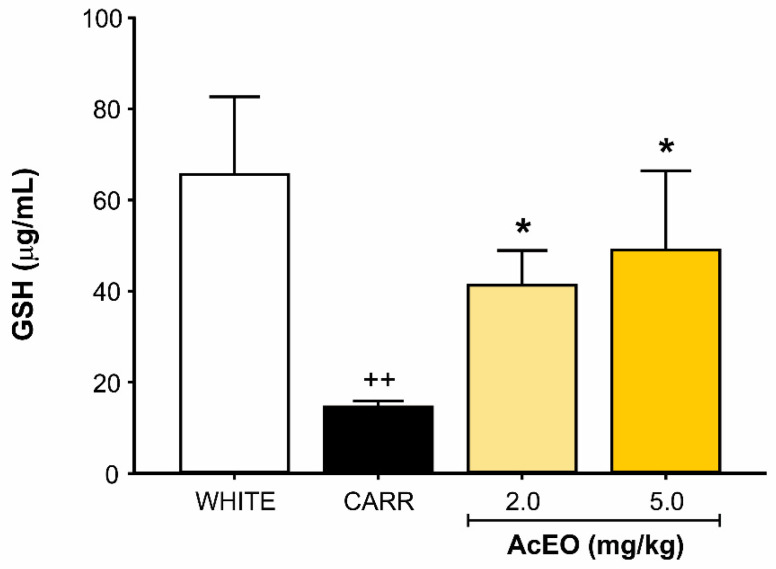
Effect of *Aniba canelilla* essential oil (AcEO; 2.0 and 5.0 mg/kg; ip.) on peritoneal fluid GSH concentration of rats submitted to carrageenan (CARR)-induced (0.3 mg/kg) peritonitis. Data expressed as mean ± SEM (*n* = 5–6/group). ^++^ *p* < 0.01 vs. white group; * *p* < 0.05 vs. CARR group. Kruskal–Wallis followed by Dunn’s test.

Carrageenan (0.3 mg/kg; ip.) promoted a significant decrease in peritoneal fluid GSH levels when compared to white group (14.59 ± 1.26 μg/mL and 65.59 ± 17.06 μg/mL, respectively; *p* = 0.002). AcEO treatment (2.0 and 5.0 mg/kg; ip.) was able to prevent GSH decrease (41.27 ± 7.67 μg/mL and 49.06 ± 17.45 μg/mL, respectively), which maintained levels equivalent to white group (*p* = 1.00 for both; Figure 4).

#### 3.2.3. AcEO Prevents Lipid Peroxidation

**Figure 5:**
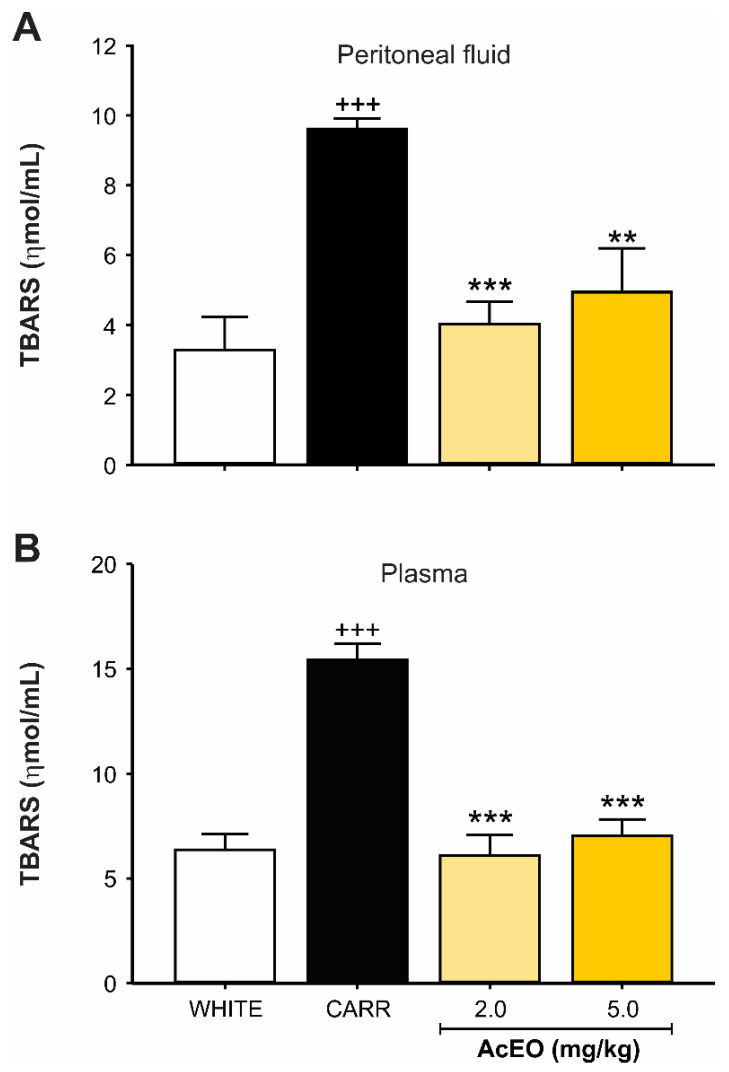
Effect of *Aniba canelilla* essential oil (AcEO; 2.0 and 5.0 mg/kg; ip.) on thiobarbituric acid reactive substances (TBARS) level in (**A**) peritoneal fluid and (**B**) plasma of rats submitted to carrageenan (CARR)-induced (0.3 mg/kg) peritonitis. Data expressed as mean ± SEM (*n* = 5–6/group). ^+++^ *p* < 0.001 vs. white group; ** *p* < 0.001 vs. CARR group; *** *p* < 0.001 vs. CARR group. One-way ANOVA followed by Dunnett’s test.

A significant increase in TBARS levels (*p* < 0.001), both in peritoneal fluid (Figure 5A) and in plasma (Figure 5B), was observed in animals that received carrageenan (0.3 mg/kg; ip.), when compared to white group (peritoneal fluid—9.61 ± 0.30 ηmol/mL and 3.29 ± 0.94 ηmol/mL, respectively; plasma—15.44 ± 0.76 ηmol/mL and 6.37 ± 0.74 ηmol/mL, respectively). On the other hand, AcEO treatment (2.0 and 5.0 mg/kg; ip.) was able to reduce TBARS concentration (peritoneal fluid–4.03 ± 0.63 ηmol/mL and 4.95 ± 1.26 ηmol/mL, respectively; plasma–6.08 ± 0.99 ηmol/mL and 7.03 ± 0.78 ηmol/mL, respectively) to white group equivalent levels (*p* < 0.001 for both; Figure 5).

### 3.3. AcEO Activity on Vascular Permeability

#### 3.3.1. AcEO Inhibits Plasma Leakage into Peritoneal Cavity

**Figure 6:**
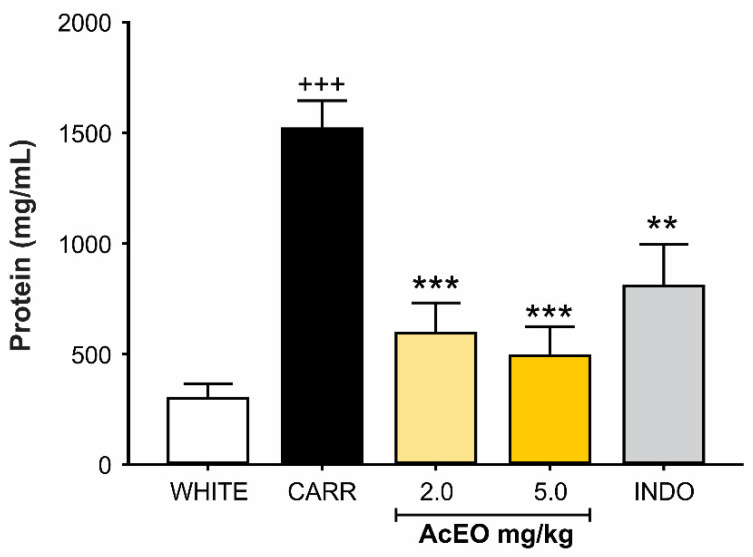
Effect of *Aniba canelilla* essential oil (AcEO; 2.0 and 5.0 mg/kg; ip.) on peritoneal fluid protein concentration of rats submitted to carrageenan (CARR)-induced (0.3 mg/kg) peritonitis. Data expressed as mean ± SEM (*n* = 5–6/group). ^+++^ *p* < 0.001 vs. white group; *** *p* < 0.001 vs. CARR group; ** *p* < 0.01 vs. CARR group. One-way ANOVA followed by Dunnett’s test.

A significant increase was observed in peritoneal fluid proteins concentration of the animals that received carrageenan (0.3 mg/kg; ip.), when compared to the white group (300 ± 65,47 mg/mL and 1517.86 ± 126.69 mg/mL, respectively; *p* < 0.001). In peritoneal fluid of animals treated with AcEO (2.0 and 5.0 mg/kg; ip.), an inhibition of this elevation was observed (592.86 ± 137.67 mg/mL and 491.07 ± 132.33 mg/kg, respectively), showing protein levels equivalent to white group (*p* = 0.385 and 0.760, respectively). The oil effect was also like non-steroidal anti-inflammatory indomethacin (*p* = 0.371 and 0.643, respectively) (Figure 6).

#### 3.3.2. AcEO Reduces Leukocyte Migration into the Peritoneal Cavity

**Figure 7:**
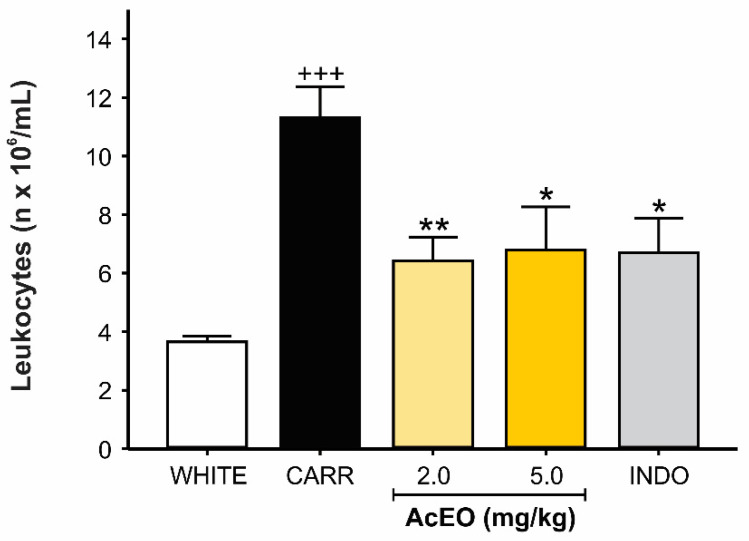
Effect of *Aniba canelilla* essential oil (AcEO; 2.0 and 5.0 mg/kg; ip.) on peritoneal fluid number of leukocytes of rats submitted to carrageenan (CARR)-induced (0.3 mg/kg) peritonitis. Data expressed as mean ± SEM (*n* = 3–5/group). ^+++^ *p* < 0.001 vs. white group; ** *p* < 0.01 vs. CARR group; * *p* < 0.05 vs. CARR group. One way ANOVA followed by Dunnett’s test.

Carrageenan administration induced a significant increase in peritoneal fluid leukocyte count (11.32 ± 1.05 × 10^6^) when compared to white group (3.94 ± 0.25 × 10^6^). Treatment with AcEO (2.0 and 5.0 mg/kg), however, inhibited carrageenan-induced leukocyte migration (6.42 ± 0.81 × 10^6^ and 6.78 ± 1.49 × 10^6^), promoting levels equivalent to those of indomethacin (6.7 ± 1.18 × 10^6^) (Figure 7).

## 4. Discussion

The present study demonstrates for the first time the effect of AcEO on oxidative balance and vascular permeability in a murine model. The research was conceived on the basis of the identification of gaps in knowledge about the pharmacological properties of *A. canelilla*, widely diffused in Amazonian medicinal culture, being used for the treatment of inflammatory, painful processes and affections of the digestive, respiratory, and central nervous systems. In a recent review developed by our group [30], the potential of this aromatic plant for the development of innovative therapeutic agents was discussed; however, knowledge about its properties is still scarce, especially with respect to its antioxidant properties, had up until that point been limited to physicochemical assays, which sought to demonstrate its ability to scavenge free radicals. There was also a lack of data regarding its effects on vascular permeability. Our findings demonstrated that AcEO has significant antioxidant activity in a stress context, in addition to modulating vascular permeability.

AcEO yield and composition can vary significantly depending on geographic region and seasonal collection period, as well as the part of the plant from which it is extracted [40]. However, studies so far have shown that EO extracted from leaves does not exhibit yield variation as a function of seasonality [41]. Taveira et al. [41] showed a yield variation between 0.5 and 0.8% in EO extracted from leaves, which was related to soil type. In our study, the EO extracted from leaves collected in May, the rainy season [42], showed a yield of 1.3%.

Regarding its composition, it has been described that EO extracted from leaves has the highest concentrations of 1N2F, ranging from 39% (dry periods) to 95% (rainy periods) [41]. Our sample, collected during the rainy season, presented a 1N2F content of 77.5%. The main bioactive AcEO properties, including anti-inflammatory and antinociceptive, were attributed to this benzenoid, recognized by its characteristic cinnamon-like odor [19,21,22,23,24,25].

Inflammation and oxidative stress are closely related mechanisms that are both part of the immune response and are also associated with the genesis and evolution of several pathologies. Therefore, considering the AcEO and its main potential component, the carrageenan-induced peritonitis model was adopted, since it induces inflammation, linked to marked oxidative stress [43,44].

The cavitary response triggered by intraperitoneal carrageenan (0.3 mg/kg) administration is marked by the phasic release of inflammatory mediators, such as histamine, serotonin, kinins and prostanoids, as well as ROS/RNS production [43,44,45]. Consequently, increases in vascular permeability and leukocyte migration and activation are observed, which also enhances the ROS/RNS production [43]. These, in turn, positively modulate the necrosis factor κB (NFκB), a key transcription factor of inflammatory response [46].

In this complex process, the excessive production of ROS, such as hydroxyl (^•^OH) and superoxide (O_2_^•−^) radicals, is triggered, together with increased nitric oxide (NO) synthesis, through the activation of induced nitric oxide synthase (iNOS). The reaction between these species can also lead to the formation of the peroxynitrite (ONOO^−^) radical, which together generate lipid peroxidation, protein carbonylation and DNA damage, contributing to cell dysfunction and death [4,5,47]

Its intensity can be measured indirectly, through the consumption of antioxidant agents, such as GSH, a thiol tripeptide (γ-L-glutamyl-L-cysteinylglycine), which protects cells from oxidative damage by interacting with and neutralizing reactive species [2,48,49]. In fact, a reduction in total peritoneal fluid antioxidant capacity of about 69% was observed, as well as a drop in GSH concentration of about 78%, in animals administered with carrageenan, evidencing the consumption of antioxidant agents due to reactive species overload.

The extent of oxidative damage generated was evaluated by measuring the secondary products of lipid hydroperoxides, such as hyroxinonenal and malonaldehyde (MDA), which are involved in mutagenic processes, atherogenic lesions and neurodegenerative disorders, by its reaction with thiobarbituric acid [3,4,11,46,47]. Animals administered carrageenan had intraperitoneal levels of these TBARS about 2.9 times higher than those in the white group. In plasma, the elevation was around 2.4 times.

In this scenario, treatment with AcEO proved to be effective for preventing the oxidative stress and consequent damages induced by carrageenan, since the animals treated with the EO showed a total antioxidant capacity statistically equivalent to the white group. Conjointly, the AcEO increased the concentration of GSH in the peritoneal fluid and reduced the levels of TBARS in peritoneal fluid and plasma, producing results equivalent to the white group (which was not administered with carrageenan). These findings demonstrate the oil’s ability to reduce oxidative stress by improving the GSH contingent and inhibiting lipid peroxidation induced by carrageenan.

Da Silva et al. (2007) [21] demonstrated, in a physical–chemical test, that the AcEO extracted from the trunk wood and 1N2F are capable of neutralizing DPPH radicals, which may indicate that effects observed in present study are related to scavenging activity of its constituents. Other minor components present in the AcEO sample, such as caryophyllene oxide (4.4%) and Linalool (2.8%), have also been described as antioxidants, as they scavenge free radicals, inhibit NO production, or increase systemic antioxidants, such as GSH [50,51], which may contribute to the observed antioxidant activity.

The neutralization of ROS could explain the preservation of GSH concentration at levels similar to the white group, since it would inhibit its consumption [52,53]. This same principle justifies the preservation of total antioxidant activity. However, possible inhibitory effects on ROS-generating enzymes, such as xanthine oxidase, or the stimulation of antioxidant enzymes, should not be ruled out.

The interface between inflammation and oxidative stress amplification, as observed in carrageenan-induced peritonitis, is closely related to increased peritoneal vascular permeability and leukocyte migration. In fact, in line with the literature, this study demonstrated an increase in peritoneal fluid proteins concentration, an indicator of an increase in plasma leakage, associated with an increase in the number of leukocytes, which, as previously described, is related to both the inflammatory mediators release through p38/MARK/NFκB myeloperoxidase way activation, as with the production of ROS [54].

Vale et al. (2013) [25] demonstrated the anti-inflammatory activity of 1N2F, reducing edema induced by croton oil, carrageenan, or dextran, pointing out the possible interaction with prostaglandin endoperoxide synthase as mechanism of action. In the present study, however, its inhibitory activity on vascular permeability and leukocyte migration was demonstrated for the first time.

The combination of anti-inflammatory and antioxidant activities highlights the potential of AcEO and its major constituent, 1N2F, for the development of innovative therapeutic agents aimed at the treatment of inflammatory, chronic and degenerative diseases associated with oxidative stress.

## 5. Conclusions

In summary, our results show that AcEO, made up mostly of 1N2F, is endowed with outstanding antioxidant activity, possibly associated, at least in part, with its ability to scavenge free radicals. Its ability to modulate vascular permeability was also exposed, inhibiting plasma leakage and leukocyte migration, demonstrating its potential for the development of natural products aimed at the treatment of inflammatory conditions associated with oxidative stress.

## Figures and Tables

**Table 1:** Chemical composition of *Aniba canelilla* essential oil.

RI_(C)_	RI_(L)_	Compounds	% *
850	850 ^a^	3*Z*-hexenol	tr
933	932 ^a^	α-pinene	0.4 ± 0.3
958	952 ^a^	benzaldehyde	0.6 ± 0.4
977	974 ^a^	β-pinene	0.4 ± 0.3
1024	1020 ^a^	*p*-cymene	0.1 ± 0.0
1028	1024 ^a^	limonene	0.2 ± 0.2
1031	1026 ^a^	1,8-cineole	0.1 ± 0.1
1041	1036 ^a^	benzene acetaldehyde	1.1 ± 0.1
1100	1095 ^a^	linalool	2.8 ± 0.7
1137	1134 ^a^	benzeneacetonitrile	0.2 ± 0.1
1190	1186 ^a^	α-terpineol	0.4 ± 0.1
1195	1195 ^a^	myrtenal	0.1 ± 0.0
**1308**	**1294 ^a^**	**1-nitro-2-phenylethane**	**77.5 ± 4.8**
1357	1356 ^a^	eugenol	0.3 ± 0.1
1377	1374 ^a^	α-copaene	0.8 ± 0.4
1408	1417 ^a^	β-longipinene	1.6 ± 0.9
1420	1417 ^a^	*E*-caryophyllene	3.6 ± 2.7
1454	1452 ^a^	α-humulene	0.4 ± 0.3
1496	1498 ^a^	α-selinene	0.1 ± 0.0
1509	1505 ^a^	β-bisabolene	0.1 ± 0.0
1525	1522 ^a^	δ-cadinene	0.1 ± 0.1
1584	1582 ^a^	caryophyllene oxide	4.4 ± 0.9
1610	1608 ^a^	humulene epoxide II	0.3 ± 0.1
1634	1639 ^a^	caryophyll-4(12),8(13)-dien-5-α-ol	0.2 ± 0.0
1637	1639 ^a^	caryophyll-4(12),8(13)-dien-5-β-ol	0.7 ± 0.1
1656	1658 ^a^	selin-11-en-4-α-ol	0.8 ± 0.0
1672	1668 ^a^	14-hydroxy-9-*epi*-*E*-caryophyllene	0.2 ± 0.2
monoterpene hydrocarbons			1.0 ± 0.9
oxygenated monoterpenes			3.5 ± 1.0
sesquiterpene hydrocarbons			6.9 ± 4.7
oxygenated sesquiterpenes			6.9 ± 1.6
**benzenoids**			**80.0 ± 5.8**
Total identified			98.4

* = mean ± standard deviation (*n* = 3); RI_(C)_ = calculated retention index on Rtx-5ms capillary column; RI_(L)_ = literature retention index, a = (Adams, 2007), tr = traces (<0.1%).

## Data Availability

Data is contained within the article.
